# Endogenous mitochondrial double‐stranded RNA is not an activator of the type I interferon response in human pancreatic beta cells

**DOI:** 10.1186/s13317-021-00148-2

**Published:** 2021-03-27

**Authors:** Alexandra Coomans de Brachène, Angela Castela, Anyïshai E. Musuaya, Lorella Marselli, Piero Marchetti, Decio L. Eizirik

**Affiliations:** 1 ULB Center for Diabetes Research, Medical Faculty, Campus Erasme, Université Libre de Bruxelles (ULB), Route de Lennik, 808-CP618, 1070 Brussels, Belgium; 2 Department of Clinical and Experimental Medicine, University of Pisa, Pisa, Italy; 3 Indiana Biosciences Research Institute, Indianapolis, IN USA

**Keywords:** Human pancreatic beta cells, Type 1 diabetes, PNPT1, Mitochondrial dsRNA, Type I interferon

## Abstract

**Background:**

Type 1 diabetes (T1D) is an autoimmune disease characterized by the progressive destruction of pancreatic beta cells. Interferon-α (IFNα), an antiviral cytokine, is expressed in the pancreatic islets in early T1D, which may be secondary to viral infections. However, not all patients harboring a type I IFN signature present signals of viral infection, suggesting that this response might be initiated by other “danger signals”. Accumulation of mitochondrial double-stranded RNA (mtdsRNA; a danger signal), secondary to silencing of members of the mitochondrial degradosome, PNPT1 and SUV3, has been described to activate the innate immune response.

**Methods:**

To evaluate whether mtdsRNA represents a “danger signal” for pancreatic beta cells in the context of T1D, we silenced PNPT1 and/or SUV3 in slowly proliferating human insulin-secreting EndoC-βH1 cells and in non-proliferating primary human beta cells and evaluated dsRNA accumulation by immunofluorescence and the type I IFN response by western blotting and RT-qPCR.

**Results:**

Only the simultaneous silencing of PNPT1/SUV3 induced dsRNA accumulation in EndoC-βH1 cells but not in dispersed human islets, and there was no induction of a type I IFN response. By contrast, silencing of these two genes individually was enough to induce dsRNA accumulation in fibroblasts present in the human islet preparations.

**Conclusions:**

These data suggest that accumulation of endogenous mtdsRNA following degradosome knockdown depends on the proliferative capacity of the cells and is not a mediator of the type I IFN response in human pancreatic beta cells.

## Background

Type 1 diabetes (T1D) is a chronic autoimmune disease characterized by the progressive destruction of pancreatic beta cells by the immune system and subsequent loss of insulin secretion. More than 50 gene variants, including several genes that regulate the antiviral response at the islet level, are associated with increased disease risk [1, 2]. Interferon-α (IFNα), a type I IFN involved in innate immunity and the antiviral response, is expressed in islets of patients affected by T1D, as first described by Foulis et al. who found IFNα in pancreases of 33/34 patients with T1D, while it was detected in only 4/80 controls [3]. More recently, analysis of laser-captured islets from five patients with recent-onset T1D showed that > 30% of the 84 IFN-stimulated genes (ISG) analysed where overexpressed by at least fivefold in these patients compared with islets from five non-diabetic control organ donors [4]. Additionally, T1D patients have higher circulating levels of IFNα than controls. Thus, Chehadeh et al. detected elevated levels of IFNα in plasma of 70% (39/56) of T1D patients and 50% of these IFNα-positive patients were also positive for enterovirus RNA, while IFNα-negative patients were negative for enterovirus RNA [5]. Accordingly, enteroviral infection of beta cells, particularly by coxsackievirus B (CVB), has been associated with T1D development [1, 6]. Viral infections may stimulate the production of IFNα in the vicinity of the beta cells and contribute to trigger autoimmunity in genetically predisposed individuals [1]. Enterovirus RNA or proteins are found in blood, stool or pancreatic islets from T1D patients more frequently than in controls [7, 8], but many patients that develop disease don’t show a clear correlation with viral infection [9], suggesting the existence of other “danger signals” that may initiate a type I IFN response in the context of T1D.

Viral replication may lead to accumulation of cytosolic double-stranded RNA (dsRNA), which is recognized by sensors such as retinoic acid-inducible gene I (RIG-I)-like receptors (RLRs), including *DDX58* (also known as *RIG-I*) and *IFIH1* (also known as melanoma differentiation-associated protein-5 *MDA5*) [10]. Other sources of dsRNAs, derived from intracellular processes such as the transcription of repetitive sequences, are also present naturally in mammalian cells. This poses a challenge to these cells, as they must discriminate between self and non-self dsRNA to avoid accidental activation of the type I IFN signaling. This discrimination is made possible by different mechanisms [11], including adenosine-to-inosine (A-to-I) editing of cellular dsRNA by the RNA-editing enzyme Adenosine deaminase RNA-specific (ADAR). Indeed, mutations in ADAR1 cause the autoimmune Aicardi-Goutières syndrome associated with a type I IFN signature [12, 13]. Another cellular source of dsRNA is the mitochondria, where the circular genome is transcribed bidirectionally and the RNA generated from the two mitochondrial DNA strands (H and L strands) can generate long mitochondrial dsRNA (mtdsRNA). The mitochondrial RNA degradosome, formed by the Suv3-like RNA helicase *SUPV3L1* (also known as *SUV3*) and the polynucleotide phosphorylase *PNPase* (also known as *PNPT1*), mediates the rapid decay of the non-coding L-strand transcripts which prevents the formation of potentially immunogenic dsRNA [14]. In line with this, depletion of PNPT1 in HeLa cells led to an abnormal accumulation of mtdsRNA, its recognition by RIG-I and MDA5, and the subsequent activation of a type I IFN response [15]. These and other similarly observations led to the suggestion that generation of mtdsRNA is an important mechanism in the triggering of antiviral signaling in humans [15].

Against this background, we presently evaluated whether generation of mtdsRNA is part of the potential endogenous “danger signals” generated in pancreatic beta cells that may contribute to trigger a local innate immune response in the context of T1D. Surprisingly, we observed that the degradosome silencing-induced mtdsRNA accumulation is a cell type specific event, present in the human insulin-secreting EndoC-βH1 cells and islet fibroblasts but not in primary human beta cells, suggesting that the phenomenon depends on the proliferative status of cells. In addition, abnormal accumulation of mtdsRNA does not induce a type I IFN signature in human pancreatic beta cells, indicating that this is not a direct contributory factor for T1D initiation.

## Methods

### Culture of human EndoC-βH1 cells, HeLa cells and human islets and cell treatment

EndoC-βH1 cells, a human pancreatic beta cell line provided by Dr. R. Scharfmann (University of Paris, France) [16], were cultured in Matrigel-fibronectin-coated plates as previously described [17]. These are slowly proliferating cells, dividing every 5 days [16]. EndoC-βH1 cells have been shown to be chronically infected by a xenotropic mouse retrovirus [18]. However, our RNAseq data indicate that they do not present an increased basal innate immune response and have also a similar immune response when treated with IFNα as compared to primary human islets [19]. The human cervical cancer cells HeLa were grown in Dulbecco’s modified Eagle’s medium (Lonza, Basel, Switzerland) supplemented with 10% FBS and 2% penicillin–streptomycin (Lonza). These cell lines were free of mycoplasma infection, as determined by monthly testing using the MycoAlert Mycoplasma Detection kit (Lonza).

Human islets from 6 non-diabetic organ donors (see Additional file 1) were isolated in Pisa, Italy, following a protocol previously described [20] with the approval of the local ethics committee and sent to Brussels for dispersion and experiments [21].

Each experiment considered as n = 1 corresponds to one independent biological observation, i.e. EndoC-βH1 cells and HeLa cells from different passages or human islets from different donors.

As positive controls for some of the parameters studied, the cells were treated with human IFNα (PeproTech, Rocky Hill, NJ, USA) at 2000 U/ml or transfected with 1 µg/ml of the synthetic dsRNA analog polyinosinic-polycytidylic acid (Sigma-Aldrich, Saint-Louis, MO, USA) as previously described [22].

### RNA interference

EndoC-βH1 cells or dispersed human islets were transfected overnight with 30 nmol/l of different siRNAs, the medium was then changed, and cells kept in culture for 48 h to 6 days to allow gene silencing. Transfection was performed using siRNA targeting PNPT1 (PNPT1#1: 5′-GCUGCACUACGAGUUUCCUCCUUAU-3′, PNPT1#2: 5′-CCUUUGGUGGUUGACU ACAGACAAA-3′, and PNPT1#3: 5-GGGCAGUACGAAUAGGAAUAAUUGA-3′; HSS131758, HSS131759 and HSS131760 respectively, Thermo Fisher Scientific, Waltham, MA, USA), SUV3 (5′-GGCCUCUGGACAAGAAUGAAGUAAA-3′; HSS110378, Thermo Fisher Scientific) or ADAR (5′-TTCCGTTACCGCAGGGATCTA-3′; 1027416, Qiagen, Venlo, The Netherlands) using Lipofectamine RNAiMax (Invitrogen, Carlsbad, CA, USA). In experiments with double transfection, we mixed 30 nmol/l of each siRNA used. Allstars Negative Control siRNA (siQ; Qiagen) was used as a negative control.

### Viral infection

The prototype strain of enterovirus (CVB5 / Faulkner) was obtained from the American Type Culture Collection (ATCC, Old Town Manassas, Virginia, USA). GMK cells were used to amplify viruses and their identity was confirmed by a plaque neutralization assay with CVB5 anti-sera. EndoC-βH1 cells were inoculated for 1 h at 37 °C with CVB5 at a multiplicity of infection (MOI) of 5 based on our previous studies [23]. The inoculum virus was then removed, and cells were cultured in medium containing FBS during 24 h to allow viral replication.

### mRNA extraction and real-time PCR

The Dynabeads mRNA DIRECT purification kit (Invitrogen) was used to purify polyadenylated mRNA from cultured cells, and reverse transcription was performed using the Reverse Transcriptase Core kit (RT-RTCK-03, Eurogentec, Liège, Belgium). We used the SsoAdvanced Universal SYBR Green Supermix (BIO-RAD, Hercules, CA, USA) to perform the real-time PCR amplification reactions. We generated standard curves to allow adequate quantification of our data, and corrected gene expression by the housekeeping gene β-actin, as its expression is not affected by the conditions used in this study (*data not shown*). The list of primers used in this study can be found in Additional file 2.

### Protein extraction and western blot analysis

Cells were washed with PBS and lysed using 1X Laemmli Sample Buffer (60 mM tris(hydroxymethyl)aminomethane pH 6.8, 10% Glycerol, 1.5% Dithiothreitol, 1.5% 2-mercaptoethanol, 2% Sodium dodecyl sulfate and 0.005% bromophenol blue). To confirm PNPT1 and ADAR knockdown, we used a rabbit polyclonal anti-PNPT1 antibody diluted 1/2000 (ab96176, Abcam, Cambridge, UK) and a rabbit monoclonal anti-ADAR1 antibody diluted 1/1000 (#14175, Cell Signaling Technologies, Danvers, MA, USA). To evaluate the type I IFN activation, we measured STAT1 and STAT2 phosphorylation using rabbit anti-phospho antibodies (pSTAT1: #9167 and pSTAT2: #8841, Cell signaling Technologies) diluted 1/1000, total STAT1 and STAT2 expression using a rabbit anti-STAT1 (sc-346, Santa Cruz Biotechnology, Dallas, USA) and a monoclonal rabbit anti-STAT2 (#72604, Cell Signaling Technologies) antibody, both diluted 1/1000. We have previously shown that total STAT1 and STAT2 expression are up-regulated in beta cells following exposure to a type I IFN [19]. We normalized our data for the expression of the housekeeping gene β-actin using a polyclonal rabbit anti-β-actin antibody diluted 1/2000 (#4967, Cell Signaling Technologies). A secondary donkey anti-rabbit antibody coupled with the horseradish peroxidase (711-036-152, Jackson ImmunoResearch Laboratories, Wes Grove, PA, USA) was used to detect the signal. Immunoreactive bands were visualized using the SuperSignal West Femto chemiluminescent substrate (Thermo Fisher Scientific), detected using ChemiDoc XRS + (BIO-RAD), and quantified with the Image Studio Lite v5.2 software (LI-COR Biosciences).

### Immunofluorescence

After different periods post-siRNA transfection or viral infection, cells were fixed with PFA 4% for 15 min followed by permeabilization with Triton X-100 0.3% for 10 min. Cells were blocked in PBS-BSA 3%—Tween20 0.02% during 30 min and then incubated overnight with the monoclonal mouse J2 anti-dsRNA antibody diluted 1/200 in blocking buffer (10010200, Scicons, Hungary). Cells infected with CVB5 were stained for the viral capsid protein VP1 using the enterovirus-specific polyclonal antiserum KTL-510 [24]. In dispersed human islets, beta cells were also stained for insulin using a polyclonal guinea pig anti-insulin ready-to-use antibody (IR00261-2, Agilent, Santa Clara, CA, USA) and fibroblasts were stained using the rabbit polyclonal anti-vimentin antibody diluted 1/200 (ab137321, Abcam). The signal was detected using Alexa Fluor 568 rabbit anti-mouse, Alexa Fluor 488 goat anti-guinea pig and Alexa Fluor 647 donkey anti-rabbit (A11061, A11073 and A31573 respectively, Thermo Fisher Scientific) secondary antibodies at 1/200. Hoechst 33342 (Sigma-Aldrich, Saint-Louis, MO, USA) was used for nuclear staining. The coverslips were mounted with the Glycergel mounting medium (C056330-2, Agilent) and immunofluorescence was visualized using a Zeiss microscope equipped with a camera (Zeiss-Vision, Munich, Germany). Images were acquired at ×40 magnification and analysed with the AxioVision software.

### Statistical analysis

Data are expressed as means ± SEM. A normality test was performed to evaluate the distribution of the observed determinations. Paired or unpaired *t* test were performed to assess statistical significance and results with *p* < 0.05 were considered statistically significant.

## Results

### PNPT1 silencing, alone or together with ADAR silencing, does not induce dsRNA accumulation or a type I IFN response in EndoC-βH1 cells

**Fig. 1:**
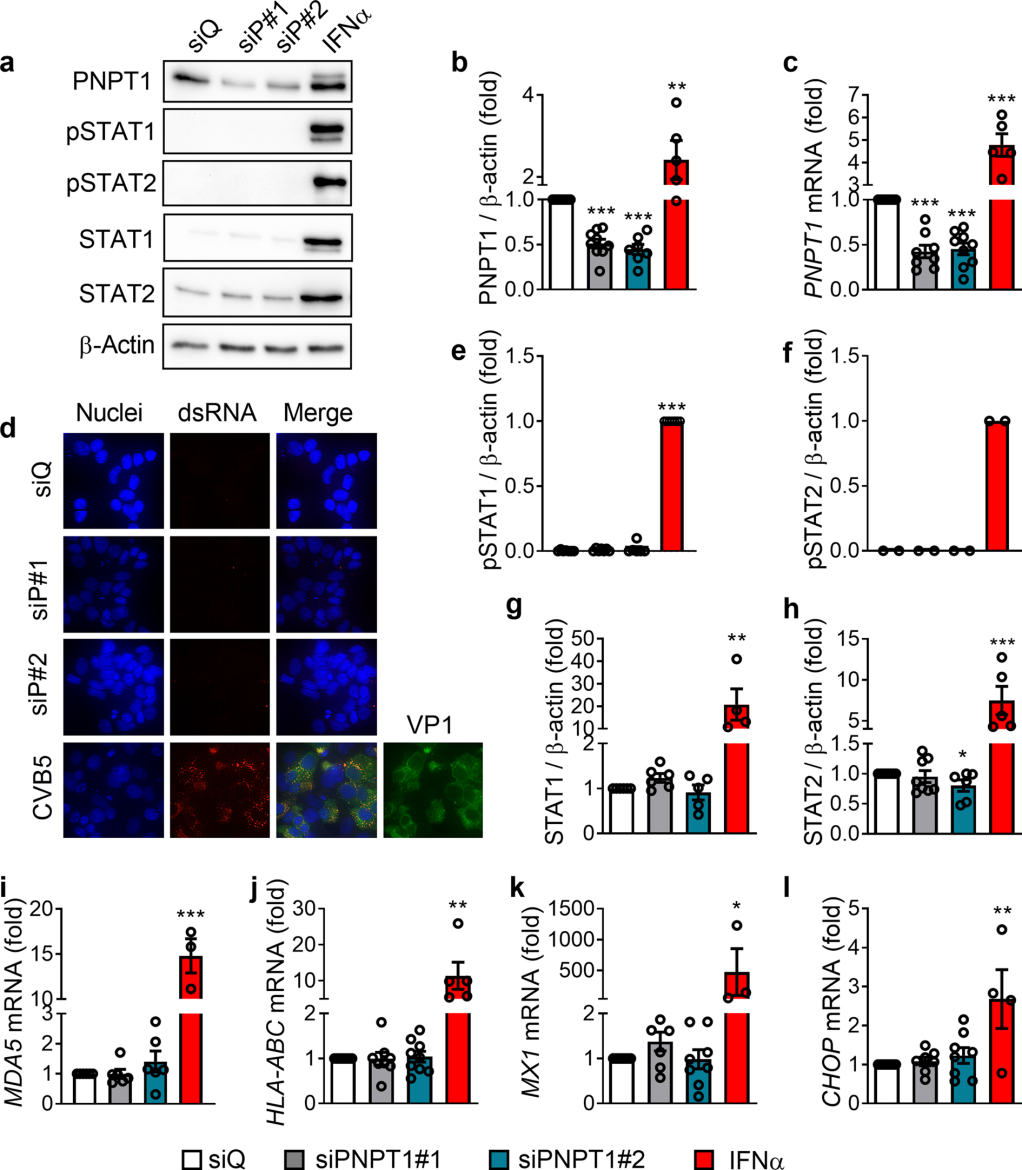
PNPT1 silencing does not induce dsRNA accumulation and a type I IFN response in EndoC-βH1 cells. EndoC-βH1 cells were transfected with a siRNA control (siQ: white bars) and two different siRNAs targeting PNPT1 (siP#1: grey bars and siP#2: blue bars) and cells were maintained in culture during 48 h. As a positive control for gene expression, in some experiments cells were treated with IFNα (2000 U/ml) for 24 h (red bars), and as a positive control for dsRNA staining, cells were infected with CVB5 (MOI 5). **a** Protein expression was measured by western blotting and representative images of 2–9 independent experiments are shown. Densitometry results are shown for PNPT1 (**b**), pSTAT1 (**e**), pSTAT2 (**f**), STAT1 (**g**) and STAT2 (**h**). **d** dsRNA accumulation (red) and the presence of the viral capside protein VP1 (green) for CVB5-infected cells was analyzed by immunocytochemistry. Representative images of 2 (siPNPT1#1) or 3 (siPNPT1#2) independent experiments and images of 1 experiment with CVB5 infection are shown (magnification 40×). mRNA expression of *PNPT1* (**c**), *MDA5* (**i**), *HLA-ABC* (**j**), *MX1* (**k**) and *CHOP* (**l**) were analyzed by RT-qPCR and normalized by β-actin and then by the value of siQ considered as 1. Results are mean ± SEM of 3–9 independent experiments. *p < 0.05, **p < 0.01 and ***p < 0.001 vs siQ, Student *t* test

To study the possible role of PNPT1 and mtdsRNA in the initiation of the innate immune response in pancreatic beta cells, as previously described in HeLa cells [15], we used two different siRNAs (siPNPT1#1 and #2). These siRNAs reduced PNPT1 expression by > 50% at both protein (Fig. 1a, b) and mRNA (Fig. 1c) levels in EndoC-βH1 cells. After 48 h of silencing, no accumulation of dsRNA was detected (Fig. 1d), while cells infected with the coxsackievirus CVB5, used as a positive control, had a strong signal for dsRNA in cells positive for the viral capside protein VP1 (Fig. 1d). Furthermore, PNPT1 knockdown did not induce a type I IFN response in EndoC-βH1 cells, as measured by the phosphorylation of STAT1 and STAT2 (Fig. 1a, e, f) and by the expression of key downstream targets of IFNα signaling, namely total STAT1 and STAT2 (Fig. 1a, g, h) at the protein level, and *MDA5* (Fig. 1i), the Human Leukocyte Antigen-ABC (*HLA-ABC* also known as Major Histocompatibility complex class I, MHC class I) (Fig. 1j), the antiviral MX dynamin-like GTPase 1 (*MX1*) (Fig. 1k) and the ER stress marker CCAAT/enhancer-binding protein homologous protein (*CHOP*, also known as *DDIT3*) (Fig. 1l) at the mRNA level. We used IFNα as a positive control since we previously showed that it induces a clear up-regulation of these genes in EndoC-βH1 cells [19, 25]. On the other hand, a clear accumulation of dsRNA was observed in the fast proliferating HeLa cells following knockdown of PNPT1 with three different siPNPT1 (#1, #2 and #3) as previously described [15] (Additional file 3a). Surprisingly, and in spite of the fact that siPNPT1#2 and #3 induced a more marked inhibition of PNPT1 expression than that observed with siPNPT1#1 (the siRNA used by Dhir et al., [15]) (Additional file 3b), only siPNPT1#1 triggered an antiviral response, as determined by the expression of *IFNβ* and *MDA5* (Additional file 3c, d).

**Fig. 2:**
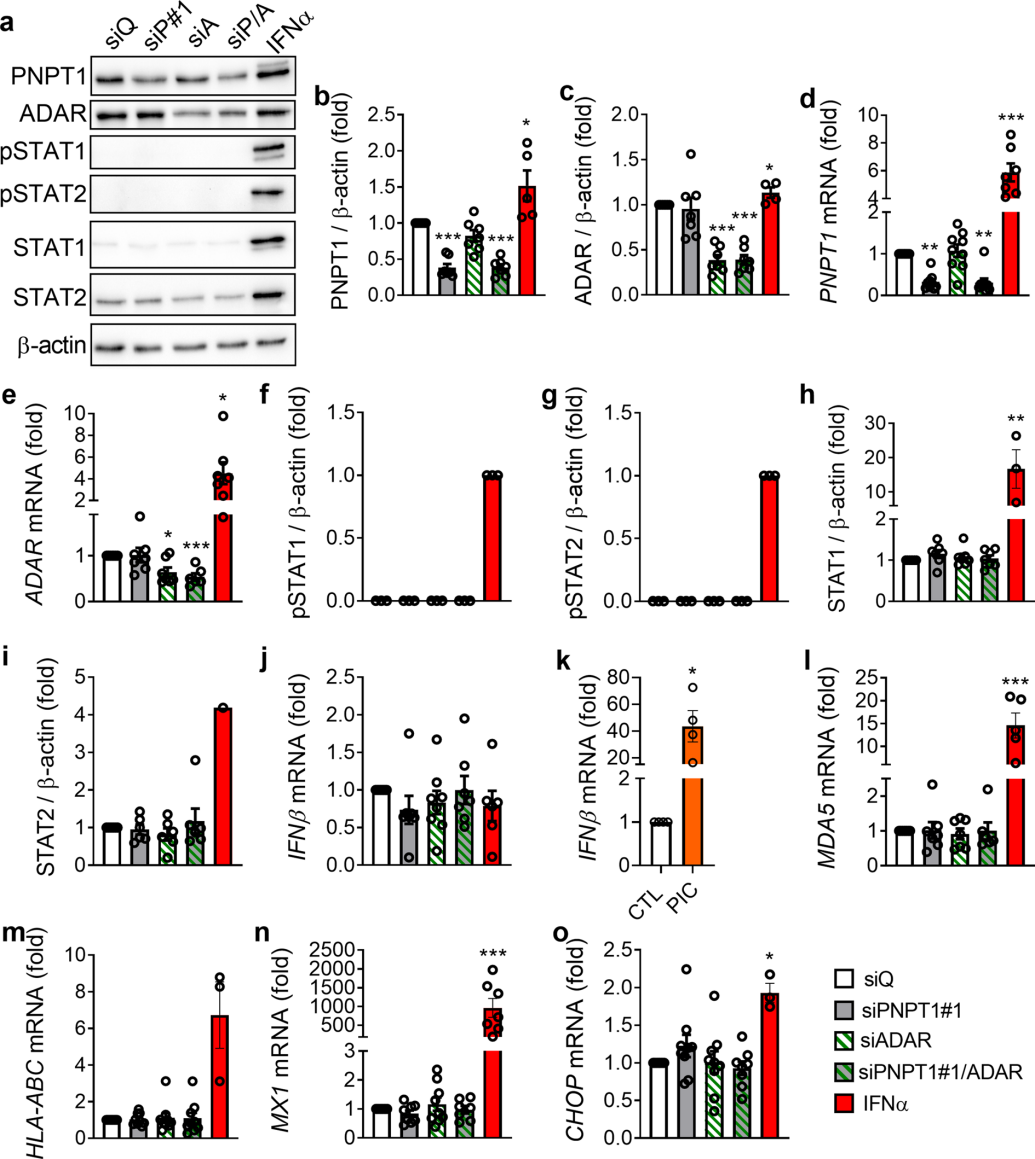
PNPT1/ADAR double silencing does not induce a type I IFN response in EndoC-βH1 cells. EndoC-βH1 cells were transfected with a siRNA control (siQ: white bars) and siRNAs targeting PNPT1 (siP#1, grey bars), ADAR (siA, green striped bars), or both (siP/A: grey bars with green stripes) and cells were maintained in culture during 48 h. As a positive control for gene expression, in some experiments cells were treated with IFNα (2000 U/ml) for 24 h (red bars), and as a positive control for *IFNβ* expression, cells were transfected with PIC (1 µg/ml) for 24 h (k, orange bar). **a** Protein expression was measured by western blotting and representative images of 3–7 independent experiments are shown. Densitometry results are shown for PNPT1 (**b**), ADAR (**c**), pSTAT1 (**f**), pSTAT2 (**g**), STAT1 (**h**) and STAT2 (**i**). mRNA expression of *PNPT1* (**d**), *ADAR* (**e**), *IFNβ* (**j**, **k**), *MDA5* (**l**), *HLA-ABC* (**m**), *MX1* (**n**) and *CHOP* (**o**) were analyzed by RT-qPCR and normalized by β-actin and then by the value of siQ considered as 1. Results are mean ± SEM of 3–9 independent experiments. *p < 0.05, **p < 0.01 and ***p < 0.001 vs siQ, Student *t* test

Next, to assess whether PNPT1 silencing-mediated mtdsRNA accumulation was masked by a rapid editing of these dsRNAs by ADAR, we silenced PNPT1 and ADAR alone or in combination in EndoC-βH1 cells, reducing their expression by > 50% at the protein (Fig. 2a–c) and mRNA (Fig. 2d–e) levels. However, the double silencing of PNPT1/ADAR neither induced accumulation of dsRNA (*data not shown*) nor activated a type I IFN response, as evaluated by the phosphorylation of STAT1 and STAT2 (Fig. 2a, f, g), and by the expression of total STAT1 and STAT2 (Fig. 2a, h, i) at the protein level, and *IFNβ* (Fig. 2j, k), *MDA5* (Fig. 2l), *HLA-ABC* (Fig. 2m), *MX1* (Fig. 2n) and *CHOP* (Fig. 2o) at the mRNA level. IFNα was used as a positive control for all genes except for *IFNβ*, where we used the synthetic dsRNA analog polyinosinic-polycytidylic acid (PIC) since IFNα does not induce its expression (Fig. 2k). Altogether, these data suggest that the silencing of PNPT1 for 48 h does not induce accumulation of immunogenic mtdsRNA in EndoC-βH1 cells.

### Long-term (6 days) double silencing of PNPT1 and SUV3 induces dsRNA accumulation in EndoC-βH1 cells but not a type I IFN response

**Fig. 3:**
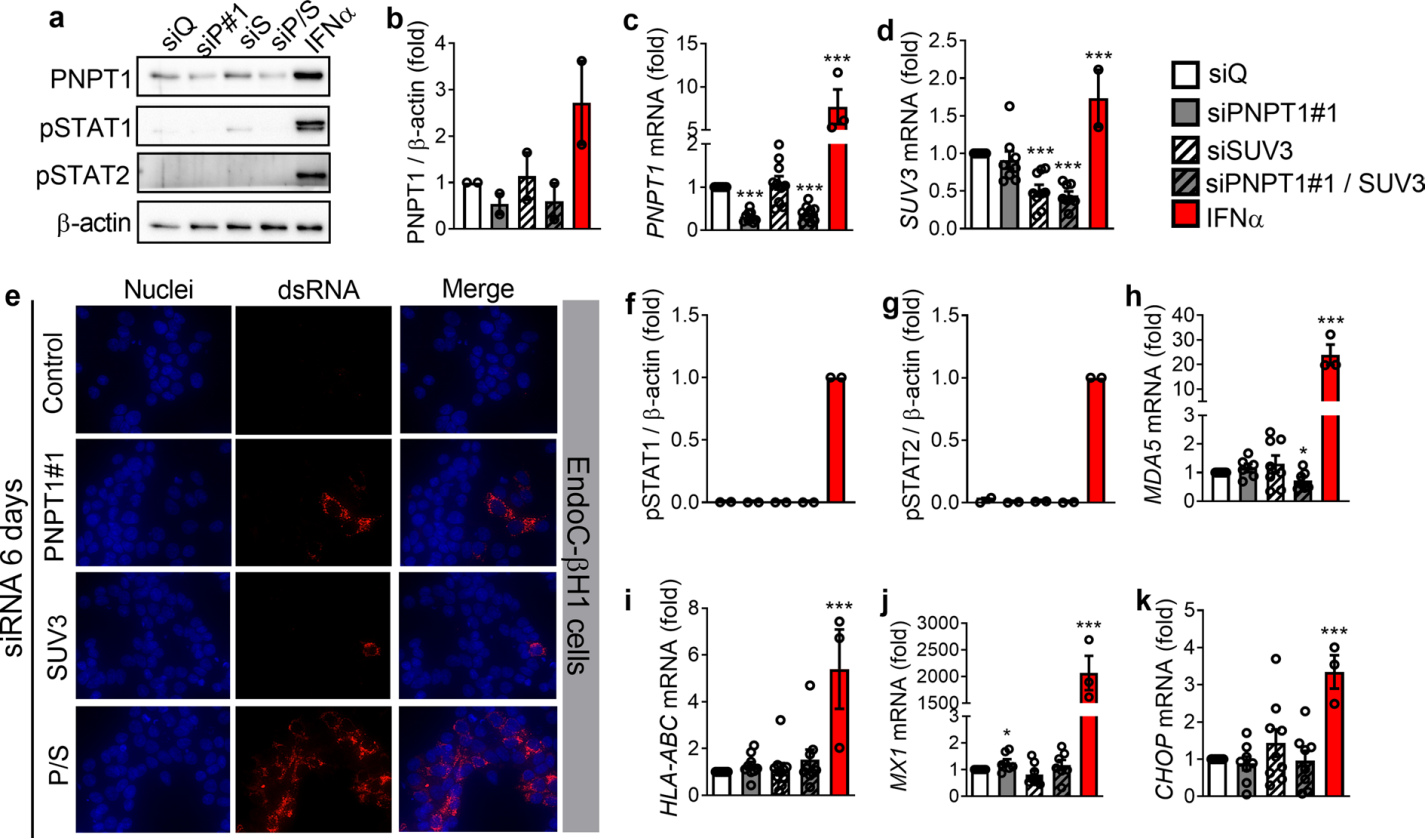
Long-term PNPT1/SUV3 silencing induces dsRNA accumulation in EndoC-βH1 cells but no type I IFN response. EndoC-βH1 cells were transfected with a siRNA control (siQ: white bars) or with siRNAs targeting PNPT1 (siP#1, grey bars), SUV3 (siS, black striped bars) or both (siP/S: grey bars with black stripes), and then maintained in culture for 6 days after transfection. As a positive control for gene expression, in some experiments cells were treated with IFNα (2000 U/ml) for 24 h (red bars). **a** Protein expression was measured by western blotting and representative images of 2 independent experiments are shown. Densitometry results are shown for PNPT1 (**b**), pSTAT1 (**f**) and pSTAT2 (**g**). **e** dsRNA accumulation (red) was analyzed by immunocytochemistry. Representative images of 3 independent experiments are shown (magnification 40×). mRNA expression of *PNPT1* (**c**), *SUV3* (**d**), *MDA5* (**h**), *HLA-ABC* (**i**), *MX1* (**j**) and *CHOP* (**k**) were analyzed by RT-qPCR and normalized by β-actin and then by the value of siQ considered as 1. Results are mean ± SEM of 2–10 independent experiments. *p < 0.05 and ***p < 0.001 vs siQ, Student *t* test

To evaluate the possible compensation of PNPT1 silencing by SUV3, the other member of the mitochondrial degradosome, we silenced these two genes simultaneously in EndoC-βH1 cells but again did not observe dsRNA accumulation after 48 h and 72 h of knockdown (*data not shown*). On the other hand, a prolonged exposure to these 2 siRNAs for 4 additional days after the usual 48 h of silencing (6 days in total) significantly reduced the expression of both PNPT1 and *SUV3* (Fig. 3a–d) and induced a clearly detectable accumulation of dsRNA in EndoC-βH1 cells (Fig. 3e). These data were confirmed by using a second siPNPT1 (Additional file 4a–c). Of note, the single knockdown of either PNPT1 or SUV3 for 6 days only modestly induced dsRNA accumulation (Fig. 3e and Additional file 4c). Despite the presence of dsRNA in EndoC-βH1 cells, there was still no induction of a type I IFN response as indicated by the absence of STAT1 and STAT2 phosphorylation (Fig. 3a, f, g) and the lack of induction of *MDA5* (Fig. 3h and Additional file 4d), *HLA-ABC* (Fig. 3i and Additional file 4e), *MX1* (Fig. 3j) and *CHOP* (Fig. 3k and Additional file 4f) expression. IFNα was used as a positive control. These findings support the importance of the collaborative action of the two members of the mitochondrial degradosome, PNPT1 and SUV3, in preventing accumulation of mtdsRNA in pancreatic beta cells. However, and different from observations in other fast-proliferating cell lines [15], in human pancreatic beta cells these mtdsRNA do not trigger a type I IFN response.

### The double silencing PNPT1/SUV3 induces dsRNA accumulation in fibroblasts but not in dispersed human islet cells

**Fig. 4:**
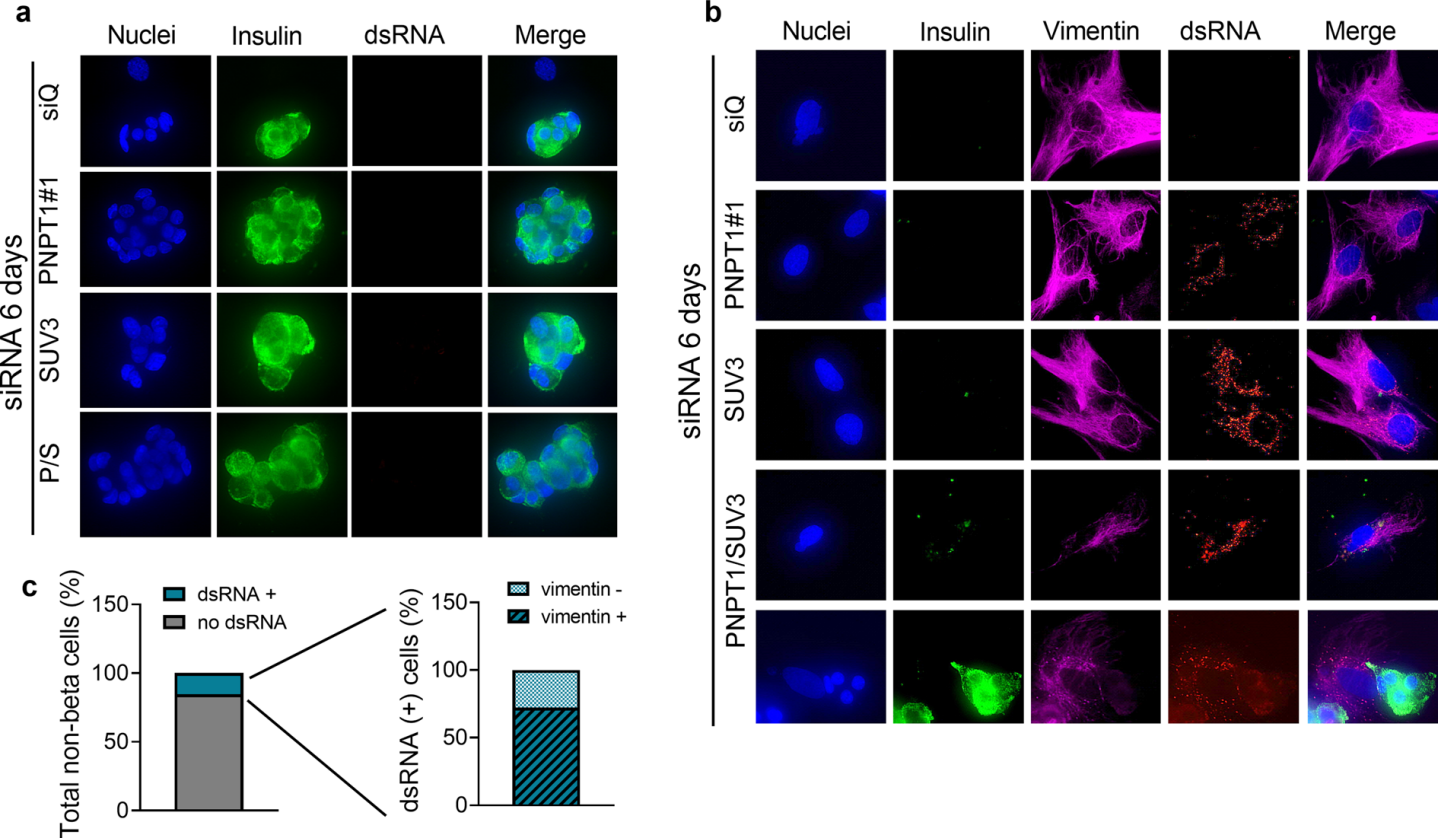
PNPT1/SUV3 knockdown induces dsRNA accumulation in surrounding fibroblasts but not in dispersed human islet cells. Dispersed human islets were transfected with a siRNA control (siQ) or with siRNAs targeting PNPT1 (#1), SUV3 or both (P/S), and then maintained in culture for 6 days after transfection. dsRNA accumulation (red), insulin content for beta cell staining (green), and vimentin for fibroblast staining (purple) were analyzed by immunocytochemistry. Representative images of 4 (**a**) and 2 (**b**, **c**) independent experiments are shown (magnification 40×). **c** dsRNA and vimentin positivity were evaluated by counting 1400 non-beta cells from 69 images obtained from 2 independent experiments

In dispersed human islets, PNPT1 and SUV3 double knockdown for 6 days (the usual 48 h of silencing followed by 4 additional days) did not induce dsRNA accumulation in the primary non-proliferating insulin-positive beta cells (Fig. 4a) as it was observed in EndoC-βH1 cells. There was, however, a clear accumulation of dsRNA in some, but not all, vimentin-positive fibroblasts present in the human islet preparations (Fig. 4b, c and Additional file 5, upper part). In a lesser proportion, other non-beta cells negative for vimentin also contained dsRNA (Fig. 4c and Additional file 5, lower part). This distinct effect, occurring in cells present in the same preparation and treated under the same experimental conditions, highlights the fact that this phenomenon is cell-type dependent and probably related to the proliferative capacity of the cells (*i.e.* fibroblasts are fast proliferating cells, while primary human beta cells practically don’t proliferate under basal culture conditions).

## Discussion

In the present study, while searching for new triggers of innate immunity in the context of T1D, we did two major observations: (1) the accumulation of mtdsRNA upon degradosome silencing seems to be cell-type specific; and (2) these mtdsRNA do not trigger a type I IFN response in human pancreatic beta cells.

In a previous study, and confirmed by our own data (Additional file 3), PNPT1 or SUV3 knockdown induced a rapid and massive accumulation of dsRNA in the fast-proliferating HeLa cells [15]. However, in slowly proliferating EndoC-βH1 cells PNPT1 silencing for 48 h did not induce dsRNA accumulation. This cannot be explained by ADAR editing as a compensatory mechanism for the inhibition of PNPT1, since the double knockdown of PNPT1 and ADAR for 48 h also failed to induce dsRNA. On the other hand, long-term (6 days) double knockdown of the two members of the mitochondrial degradosome, PNPT1 and SUV3, induced dsRNA accumulation in the slowly proliferating EndoC-βH1 cells but not in the non-proliferating primary human beta cells. Surprisingly, and confirming the cell-specific nature of this process, the vimentin-positive fibroblasts present in the human islet preparations stained positively for dsRNA after silencing of PNPT1 and/or SUV3. These data point to a cell-specific effect that could be connected to the proliferative status of the cells, as this is the main difference between EndoC-βH1 cells and primary human islet cells. Of note, we have recently shown that EndoC-βH1 cells or primary human islets treated with IFNα have a similar gene signature as compared to beta cells obtained from patients affected by T1D [19].

In line with the hypothesis that mtdsRNA accumulation correlates with the proliferative status of the cells, when studying mitochondrial RNA (mtRNA) extracted from S or M phase-arrested HeLa cells, the largest fraction of mtRNA isolated in the M phase were present as duplex RNA [26]. In addition, the analysis of mitochondrial DNA (mtDNA) replication and transcription during the cell cycle showed a clear coordination between mtDNA and nuclear DNA synthesis [27] that may explain the presently observed absence of mtdsRNA in the non-proliferating primary beta cells.

We also noticed that mtdsRNA accumulation in EndoC-βH1 cells fail to induce a type I IFN response, different from previous observations in HeLa cells [15]. On the other hand, when using the same siPNPT1 (siPNPT1#1) used in the study by Dhir and colleagues [15] we were able to reproduce the observed mtdsRNA accumulation in HeLa cells (Additional file 3). However, two other siPNPT1 that inhibited PNPT1 by > 70% (#2 and #3) failed to trigger the type I IFN response, in spite of inducing a clear accumulation of dsRNA in HeLa cells (Additional file 3). Thus, these data, and the data presented by Dhir and colleagues [15] using one of the presently tested siRNAs, need to be taken with caution as we can’t exclude that some of the effects observed with siPNPT1#1 are off target effects, unrelated from the actual inhibition of PNPT1. In any case, none of these siPNPT1, alone or combined with SUV3 siRNA, induced a type I IFN response in EndoC-βH1 cells, despite the presence of dsRNA. This indicates that mtdsRNA accumulating upon degradosome inactivation is probably not a mediator of the type I IFN response in human pancreatic islets.

## Conclusions

Our findings support the concept that accumulation of mtdsRNA following mitochondrial degradosome knockdown is not a general cell mechanism but is preferentially present in fast proliferating cells. More importantly, accumulation of mtdsRNA is not a general trigger of the innate immune response, as it fails to do so in human pancreatic beta cells. Therefore, mtdsRNA does not seem to be an endogenous “danger signal” for the induction of IFNα in the beta cells in the context of T1D. We cannot exclude, however, an indirect effect of these mtdsRNA in the development of T1D through their release from the beta cells via exosomes, and their subsequent action on immune cells as described in the context of alcoholic liver disease [28].

## Supplementary Information

**Additional file 1.** Characteristics of the human donors providing the islets used in the present study.

**Additional file 2.** List of primers used in the study.

**Additional file 3.** PNPT1 knockdown in HeLa cells induces dsRNA accumulation but a type I IFN response is only observed with one siRNA out of three tested.

**Additional file 4.** A second siPNPT1 confirms the accumulation of dsRNA after the double knockdown of PNPT1 and SUV3 for 6 days in EndoC-βH1 cells, without a type I IFN response.

**Additional file 5.** The double silencing PNPT1/SUV3 induces dsRNA accumulation in non-beta cells of the human islet preparations.

## Data Availability

All data generated or analysed during the current study are available from the corresponding author on reasonable request.

## Abbreviations

CVB: Coxsackievirus B
dsRNA: Double-stranded RNA
ER: Endoplasmic reticulum
IFNα: Interferon-α
IFNβ: Interferon-β
MOI: Multiplicity of infection
mtDNA: Mitochondrial DNA
mtdsRNA: Mitochondrial double-stranded RNA
PIC: Polyinosinic-polycytidylic acid
siRNA: Small-interfering RNA
T1D: Type 1 diabetes
