# Supplementary material for: Endogenous mitochondrial double‐stranded RNA is not an activator of the type I interferon response in human pancreatic beta cells
Source: Auto Immun Highlights. 2021 Mar 27;12(1):6. doi: 10.1186/s13317-021-00148-2 (PMC8005246; doi:10.1186/s13317-021-00148-2)
Supplement: Supplementary file 1 — Additional file 1. Characteristics of the human donors providing the islets used in the present study. [file 13317_2021_148_MOESM1_ESM.docx]

**Additional file**

**Additional file 1.** Characteristics of the human donors providing the islets used in the present study.

| Date of islet isolation | Age (years) | Gender | BMI (kg/m^2^) | Cause of death | Estimated purity (%) |
| --- | --- | --- | --- | --- | --- |
| 06/04/19 | 69 | F | 33.1 | PE | 31 |
| 15/04/19 | 50 | F | 25.4 | CH | 45 |
| 02/07/19 | 80 | M | 26.2 | CD | 36 |
| 12/07/19 | 83 | M | 31.1 | CD | 50 |
| 16/07/20 | 88 | M | 24.5 | CD | 68 |
| 28/07/20 | 77 | M | 24.2 | CD | 35 |

M: male; F: female; BMI: body mass index; PE: Postanoxic Encephalopathy; CH: Cerebral Hemorrhage; CD: Cardiovascular Disease. Beta cell purity (*i.e.* the % of beta cells in the prepartions) was determined by immunohistochemistry for insulin.
