# Supplementary material for: Endogenous mitochondrial double‐stranded RNA is not an activator of the type I interferon response in human pancreatic beta cells
Source: Auto Immun Highlights. 2021 Mar 27;12(1):6. doi: 10.1186/s13317-021-00148-2 (PMC8005246; doi:10.1186/s13317-021-00148-2)
Supplement: Supplementary file 2 — Additional file 2. List of primers used in the study. [file 13317_2021_148_MOESM2_ESM.docx]

**Additional file**

**Additional file 2.** List of primers used in the study.

|  | Forward (5’-3’) | Reverse (5’-3’) |
| --- | --- | --- |
| Actin | CTGTACGCCAACACAGTGCT | GCTCAGGAGGAGCAATGATC |
| PNPT1 | AAATGAGCCTGATGTCCTAGC | AGGCTTCCAACATGACAATCT |
| SUV3 | GGCCTCTGGACAAGAATGAA | ATCCTCTGGCTGGATCTCTAA |
| HLA-ABC | CAGGAGACACGGAATGTGAA | TTATCTGGATGGTGTGAGAACC |
| CHOP | Qiagen QuantiTect primer, cat# QT00082278 | |
| MX1 | AGACAGGACCATCGGAATCT | GTAACCCTTCTTCAGGTGGAAC |
| IFNβ | GTTGAGAACCTCCTGGCTAATG | GGTAATGCAGAATCCTCCCATAAT |
| MDA5 | GAGGAATCAGCACGAGGAATAA | TCAGATGGTGGGCTTTGAC |
| ADAR | GAGAGGCTTCATCAGGTTTCTCT | CTCCTCCCTTAGCAGGTTCAAAT |
