# Supplementary material for: Endogenous mitochondrial double‐stranded RNA is not an activator of the type I interferon response in human pancreatic beta cells
Source: Auto Immun Highlights. 2021 Mar 27;12(1):6. doi: 10.1186/s13317-021-00148-2 (PMC8005246; doi:10.1186/s13317-021-00148-2)
Supplement: Supplementary file 3 — Additional file 3. PNPT1 knockdown in HeLa cells induces dsRNA accumulation but a type I IFN response is only observed with one siRNA out of three tested. [file 13317_2021_148_MOESM3_ESM.docx]

**Additional file**


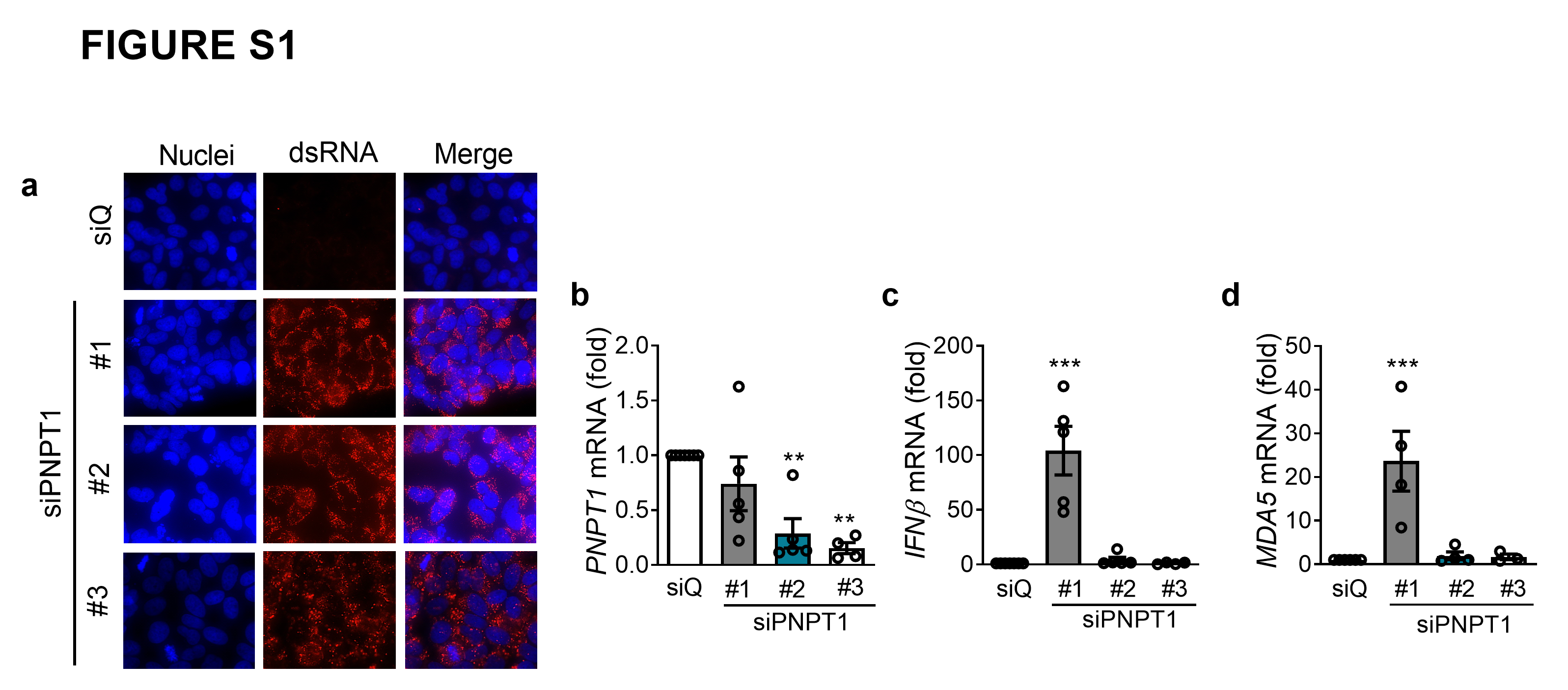


**Additional file 3. PNPT1 knockdown in HeLa cells induces dsRNA accumulation but a type I IFN response is only observed with one siRNA out of three tested**

Hela cells were transfected with a siRNA control (siQ: white bars) or with 3 different siRNAs targeting PNPT1 (#1: grey bars, #2: blue bars and #3: light grey bars) during 72h. (a) dsRNA accumulation (red) was analyzed by immunocytochemistry. Representative images of 3 (siPNPT1#1 and #2) and 2 (siPNPT1#3) independent experiments are shown (magnification 40x). mRNA expression of *PNPT1* (b), *IFNβ* (c) and *MDA5* (d) were analyzed by RT-qPCR and normalized by β-actin and then by the value of siQ considered as 1. Results are mean ± SEM of 4-5 independent experiments. ***p*<0.01 and ****p*<0.001 vs siQ, ANOVA.
