# Supplementary material for: Endogenous mitochondrial double‐stranded RNA is not an activator of the type I interferon response in human pancreatic beta cells
Source: Auto Immun Highlights. 2021 Mar 27;12(1):6. doi: 10.1186/s13317-021-00148-2 (PMC8005246; doi:10.1186/s13317-021-00148-2)
Supplement: Supplementary file 4 — Additional file 4. A second siPNPT1 confirms the accumulation of dsRNA after the double knockdown of PNPT1 and SUV3 for 6 days in EndoC-βH1 cells, without a type I IFN response. [file 13317_2021_148_MOESM4_ESM.docx]

**Additional file**


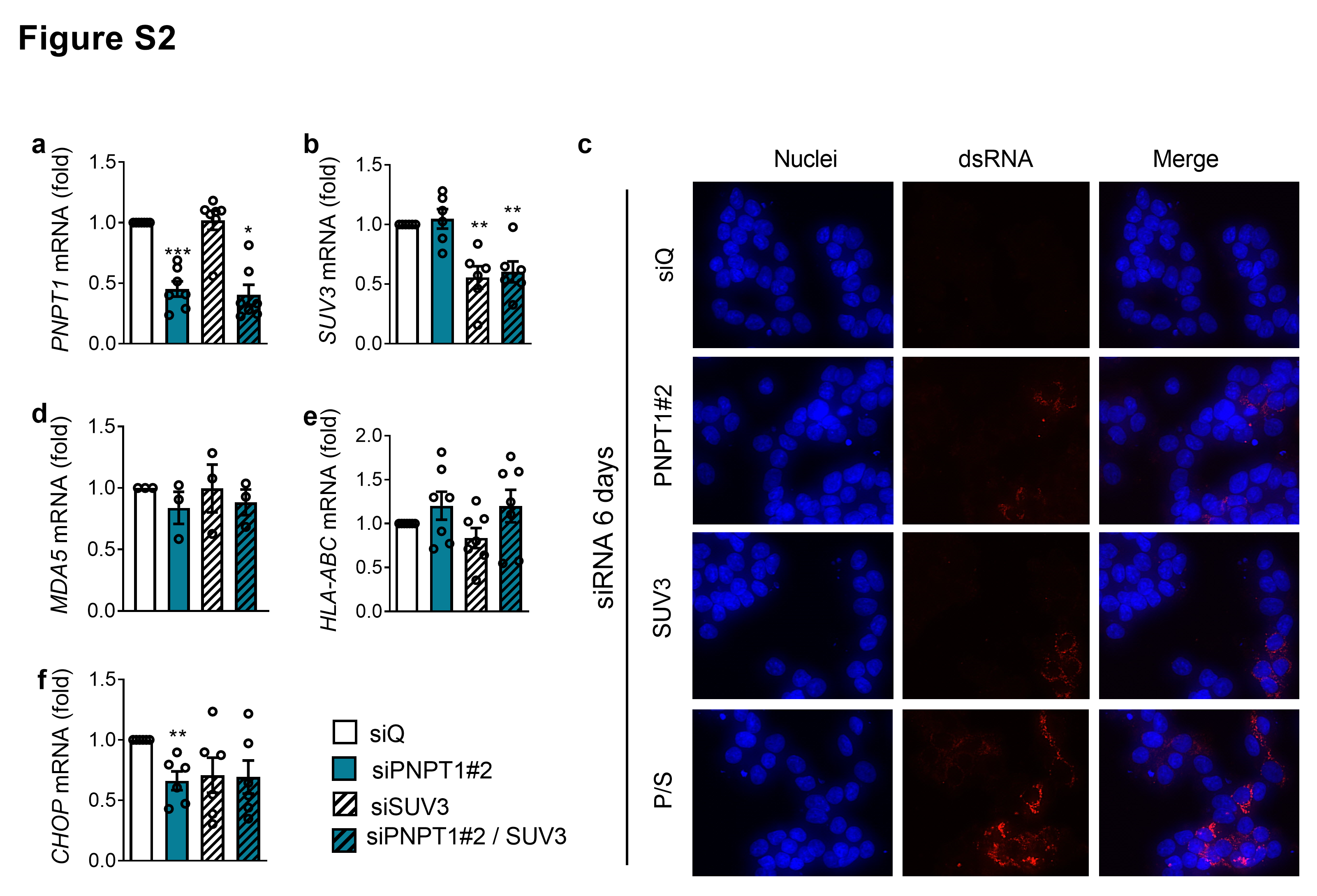


**Additional file 4. A second siPNPT1 confirms the accumulation of dsRNA after the double knockdown of PNPT1 and SUV3 for 6 days in EndoC-βH1 cells, without a type I IFN response**

EndoC-βH1 cells were transfected with an siRNA control (siQ: white bars) or with siRNAs targeting PNPT1 (#2: blue bars), SUV3 (black striped bars) or both (P/S, blue bars with black stripes), and cells were maintained in culture during 6 days after transfection. mRNA expression of *PNPT1* (a), *SUV3* (b), *MDA5* (d) *HLA-ABC* (e) and *CHOP* (f) were analyzed by RT-qPCR and normalized by β-actin and then by the value of siQ considered as 1. Results are mean ± SEM of 3-6 independent experiments. (c) dsRNA accumulation (red) was analyzed by immunocytochemistry. Representative images of 4 independent experiments are shown (magnification 40x). **p*<0.05, ***p*<0.01 and ****p*<0.001 vs siQ, Student *t* test.

.
