# Supplementary material for: Endogenous mitochondrial double‐stranded RNA is not an activator of the type I interferon response in human pancreatic beta cells
Source: Auto Immun Highlights. 2021 Mar 27;12(1):6. doi: 10.1186/s13317-021-00148-2 (PMC8005246; doi:10.1186/s13317-021-00148-2)
Supplement: Supplementary file 5 — Additional file 5. The double silencing PNPT1/SUV3 induces dsRNA accumulation in non-beta cells of the human islet preparations. [file 13317_2021_148_MOESM5_ESM.docx]

**Additional file**


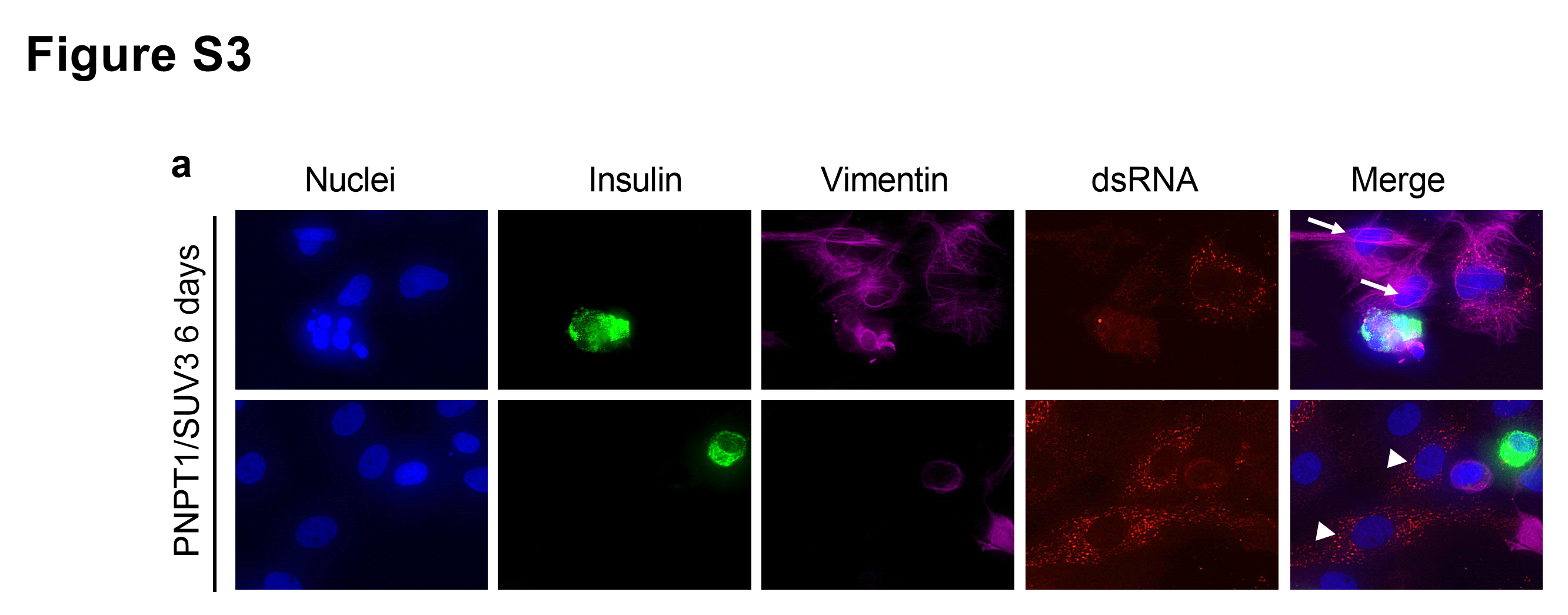


**Additional file 5. The double silencing PNPT1/SUV3 induces dsRNA accumulation in non-beta cells of the human islet preparations**

Dispersed human islets were transfected with siRNAs targeting PNPT1 (#1) and SUV3 and then maintained in culture for 6 days after transfection. dsRNA accumulation (red), insulin content for beta cell staining (green), and vimentin for fibroblast staining (purple) were analysed by immunocytochemistry. White arrows represent vimentin-positive cells without dsRNA and white arrow heads represent vimentin-negative cells containing dsRNA. Representative images of 2 independent experiments are shown (magnification 40x).
